# Enzymatic Deamidation of Plant Proteins by Protein-Glutamine Glutaminases: Effects on Solubility, Foaming and Emulsion Properties

**DOI:** 10.3390/foods15152586

**Published:** 2026-07-23

**Authors:** Nicole Roth, Diane Ebinger, Gudrun Horstmann, Lutz Fischer

**Affiliations:** Department of Biotechnology and Enzyme Science, Institute of Food Science and Biotechnology, University of Hohenheim, Garbenstr. 25, 70599 Stuttgart, Germany; nicole.roth@uni-hohenheim.de (N.R.);

**Keywords:** techno-functional properties, *Bacteroides helcogenes*, *Chryseobacterium proteolyticum*, Flavobacterium, oat protein, sunflower protein, pea protein, broad bean protein

## Abstract

Enzymatic deamidation using protein-glutamine glutaminases (PGs) is a promising method for improving the solubility, foaming properties, and emulsifying properties of plant proteins. This study compared the deamidation of various plant proteins using PGs from *Chryseobacterium proteolyticum* (PGC), *Bacteroides helcogenes* (PGB), and *Flavobacterium* sp. (PGF) and examined the effects on the foaming and emulsifying properties of sunflower and oat proteins. PGB produced the largest increases in protein solubility, though PGF usually gave the highest maximal degree of deamidation (DD(max)). For oat protein, all PGs reduced maximum foam volume (V_F_(max)), but PGC and PGB doubled foam half-time (t_1/2 foam_). For sunflower protein, PGB reduced V_F_(max), while PGC increased t_1/2 foam_ by up to 17.5-fold. Thus, different PGs affect techno-functional properties distinctly, reflecting different substrate specificities. Improvements in solubility, foaming, and emulsifying properties are not dependent on achieving the highest DD(max). Expanding the range of available PGs would allow for better matching to plant substrates and enhance industrial applicability.

## 1. Introduction

A growing world population and the demand for sustainable, nutritious food drive the search for alternative protein sources like plant proteins. Solutions are sought to exchange animal proteins with plant alternatives without changing the texture or taste of the food [1]. Although plant proteins are widely and cheaply available and show beneficial amino acid compositions, they are not yet fully utilized in the food industry, due to their low solubility in neutral aqueous systems [2] as well as poor foaming and emulsifying properties [3,4]. Interesting candidates for application in the food industry include proteins from oat (*Avena sativa* L.) and sunflower (*Helianthus annuus*), as well as different legumes like pea (*Pisum sativum* L.) and broad bean (*Vicia faba* L.). Oat protein shows low solubility, foam volume and stability as well as emulsion stability [5]. Sunflower meal contains a high protein content; however, sunflower proteins are not soluble at neutral pH, which results in poor foam volume and stability as well as poor emulsion properties [6]. Legumes like pea and broad bean also contain a high protein content and a balanced amino acid profile; however, protein solubility as well as low emulsion and foam stabilities hinder the industrial application [4,7,8]. To address the techno-functional limitations of plant proteins in the food industry, enzymatic methods, like deamidation, offer a potential solution by changing the protein conformation and surface charge [9].

Protein-glutamine glutaminases (PG, EC 3.5.1.44) catalyze the deamidation of protein-bound L-glutamine into L-glutamic acid in the presence of water, thus releasing ammonia [10,11]. The first PG was isolated from *Chryseobacterium proteolyticum* (PGC) [10] and is the only commercially available PG to date [12]. PGC is a monomeric enzyme natively expressed as a prepro-protein consisting of a signal peptide (21 amino acids) for secretion, a pro-peptide (114 amino acids) and the mature protein (185 amino acids) [13,14]. The pro-peptide is cleaved extracellularly by a peptidase resulting in the active PGC [11]. Other PGs were discovered in the genomes of *Chryseobacterium. cucumeris* [15], *Bacteroides helcogenes* (PGB) and *Flavobacterium* sp. 316 (PGF) [16,17]. Like PGC, the PGs from *B. helcogenes* and *Flavobacterium* sp. 316 also consist of a pro-sequence and a mature sequence, as well as a putative signal peptide. Like pro-PGC, pro-PGF showed no activity with the attached pro-sequence [17,18]. Interestingly, pro-PGB showed activity although the pro-sequence was still attached [16]. However, the cleavage of the pro-sequence using trypsin as an alternative for native activation further increased the activity of PGB 17.5-fold [17]. Cleaving the pro-peptide of PGC and PGF using trypsin resulted in the activation of both PGs as well, probably due to the pro-sequence cleavage in proximity to the putative native cleavage site [17,19]. Additionally, it was demonstrated that both PGC and PGB could be produced in their active form by using *Bacillus subtilis* as an expression host due to pro-peptide hydrolysis after secretion through *Bacillus*’ naturally secreted endopeptidases [19,20,21,22].

PGC has been widely used to investigate the effects of protein deamidation on the techno-functional properties of plant proteins. Foaming and emulsifying properties, as well as protein solubility, have been examined in coconut [23], evening primrose [24], oat [25], and soy protein [26], as well as in α-zein [27] and gluten [28]. While the reported effects of enzymatic deamidation on techno-functional properties varied among studies, a consistent increase in protein solubility following PGC treatment was observed. In contrast, the effects on foaming and emulsifying properties were inconsistent and depended on the protein source [23,24,25,26,27,28]. In addition, deamidation of plant proteins has been reported to positively affect the texture and flavor of food products [29,30]. The enzymatic deamidation of plant proteins increases the number of carboxyl groups, thereby increasing the net negative charge and electrostatic repulsion [31]. This increase has been linked to reduced aggregation, protein unfolding, and changes in flexibility, involving a decrease in *β*-sheets and an increase in *β*-turns, *α*-helices, and random coils [25,28,32]. In pea protein isolate, increased emulsion properties were associated with higher interfacial protein coverage and reduced aggregation [32].

This study aims to evaluate how enzymatic deamidation affects the techno-functional properties of various plant protein substrates. Therefore, the two novel PGs PGB and PGF were compared to PGC, which is commercially available and most often used in the literature. As plant protein substrates, gluten, oat, sunflower, broad bean, and pea were used. The study examined the degree of deamidation (DD) along with protein solubility to discover correlations between the plant protein source and the specific PG used. Additionally, the study analyzed the emulsion and foaming properties of the deamidated protein suspensions derived from sunflower and oat.

## 2. Materials and Methods

### 2.1. Used Chemicals

All used chemicals were at least of analytical quality and purchased from Carl Roth GmbH (Karlsruhe, Germany), Sigma-Aldrich GmbH (Taufkirchen, Germany), or Merck KGaA (Darmstadt, Germany), if not stated otherwise.

Gluten from wheat (76.0% (*w*/*w*) protein, Sigma-Aldrich Chemie GmbH, Taufkirchen, Germany), oat protein powder (30.8% (*w*/*w*) protein, n.d.), sunflower protein powder (50.0% (*w*/*w*) protein, n.d.), broad bean protein powder (73.0% (*w*/*w*) protein, Herba Ingredients BV, Wormer, The Netherlands), pea protein isolate (PPI, 86.0% (*w*/*w*) protein, n.d.), and pea protein concentrate (PPC, 55.0% (*w*/*w*) protein, Herba Ingredients BV, Wormer, The Netherlands) were used. Carbohydrate and fat concentrations are given in Supplemental Table S1.

The commercial PG preparation from *C. proteolyticum* (Amano Enzyme Inc., Nagoya, Japan) was used as a reference in this work and is abbreviated as PGC.

### 2.2. Production of Pro-PGB in Escherichia coli BL21(DE3)

The PG from *Bacteroides helcogenes* (PGB, UniProt ID: E6SWX4) was recombinantly produced in *E. coli* BL21(DE3) as described by Horstmann et al. [16] in a bioreactor. Briefly, the construct pET20b_BHPG_His10 was transformed in *E. coli* BL21(DE3), and two consecutive precultures (10 mL and 100 mL, Lysogeny broth with 100 µg mL^−1^ ampicillin) were inoculated and cultivated for 10 h at 37 °C. The production was done in Multifors bioreactors (Infors AG, Bottmingen, Switzerland) in a working volume of 0.8 L 2YT medium (tryptone, 16 g L^−1^; yeast extract, 10 g L^−1^; NaCl, 5 g L^−1^) containing 3% (*w*/*v*) glucose and ampicillin (100 µg mL^−1^) was used. The pH during cultivation was 7.0 ± 0.1, controlled by the addition of 2 M NaOH and 2 M H_3_PO_4_. The air flow was set to 1 vvm and the concentration of dissolved O_2_ (pO_2_) in the medium was kept above 20% by regulating the stirrer speed (400–800 rpm). Samples were taken regularly, and the optical density (OD_600_), bio dry mass and glucose concentration were analyzed as described before [16]. Gene expression was induced with 0.5 mM IPTG at an OD_600_ of 5 and the temperature was reduced to 30 °C. Additionally, 10 mL samples were taken regularly during cultivation and centrifuged (13,000× *g*, 15 min, 4 °C). The cell pellet was suspended in 0.9% (*w*/*v*) NaCl and centrifuged again. Afterwards, the cell pellet was stored at −20 °C until cell disruption. For cell disruption, the cell pellet was suspended in 2-(*N*-morpholino)ethanesulfonic acid buffer (MES, 100 mM, pH 5.5) and disrupted by sonification (UP200S ultrasonic processor, Hielscher Ultrasonics GmbH, Teltow, Germany, 10 cycles containing 1 min disruption and 1 min break, 95% amplitude) on ice. The cell debris was removed by centrifugation (13,000× *g*, 15 min, 4 °C) and the cell-free supernatant was used for determination of PG activity, protein content according to Bradford [33] and sodium dodecyl sulfate polyacrylamide gel electrophoresis (SDS-PAGE) as described by Horstmann et al. [16].

### 2.3. Trypsin Hydrolysis of Pro-PGB and Purification of Mature PGB

Trypsin hydrolysis was chosen for the pro-peptide cleavage of pro-PGB as described by Horstmann et al. [17] with modifications. Trypsin activity was determined according to Horstmann et al. [17] using the synthetic substrate H-Lys-*p*NA (Bachem Holding AG, Bubendorf, Switzerland). Briefly, the assay was done in a total volume of 250 µL using 50 mM sodium potassium phosphate buffer (pH 7.0) at 37 °C. The reaction was stopped by adding 50 µL 80% (*v*/*v*) acetic acid and the absorption of the released *para*-nitroaniline (*p*NA) was measured at 405 nm. One katal (kat) of trypsin activity was defined as the production of 1 mol *p*NA per second.

From the harvested cells, a 30% (*w*/*v*) cell suspension was prepared in sodium potassium phosphate buffer (50 mM, pH 7.0) and disrupted as described above. After centrifugation, the supernatant was diluted 1:2 in sodium potassium phosphate buffer and the cell debris was used to prepare a 21% (*w*/*v*) suspension in sodium potassium phosphate buffer. Both the supernatant and the suspended cell debris pellet were treated using 0.125 nkat_H-Lys-_*_p_*_NA_ mL^−1^ trypsin from bovine pancreas (AppliChem GmbH, Darmstadt, Germany) for 3 h at 37 °C. Samples were centrifuged (10,000× *g*, 10 min, 4 °C) and the supernatants were combined. Fractionated ammonium sulfate precipitation was chosen as a first purification step. A saturated ammonium sulfate solution (4 M) was used to adjust the saturation to 20%, and the sample was incubated on ice for 1 h. After centrifugation (10,000× *g*, 10 min, 4 °C), the ammonium sulfate saturation in the supernatant was raised to 55%, incubated on ice for 1 h and centrifuged as described above. The pellet after centrifugation, containing the PGB and trypsin, was suspended in binding buffer (BB, 100 mM 3-(*N*-morpholino)propanesulfonic acid (MOPS), 150 mM NaCl, pH 7.9), desalted to BB using PD-10 columns (cytiva Germany GmbH, Dreieich, Germany), filtered (0.45 µm), and applied to Ni-affinity chromatography as described by Horstmann et al. using a Takara column packed with 10 mL His60 Superflow resin (Takara Bio Inc., Kusatsu, Japan) to separate the pro-peptide and trypsin from the mature PGB [16].

### 2.4. Production of Mature PGF in B. subtilis 007

The PG from *Flavobacterium* sp. 316 (PGF; UniProt ID: A0A0D6TQX4; RefSeq: JYGZ01000001; ProteinID: KIX22659.1, gene sequence, see Supplemental Figure S1) was recombinantly produced in the *B. subtilis* wild-type strain 007 (DSM No. 118688) using the method described by Horstmann et al. for secretory PGB production [21] in baffled shake flasks. The native pro-pgf gene was synthesized by Thermo Fisher Scientific (Waltham, MA, USA), and restriction sites for *BssH*II at the 5′ end and *Xho*I at the 3′ end were inserted by polymerase chain reaction (primers, see Supplemental Table S2). DNA was purified using the DNA Clean and Concentrator kit (Zymo Research Europe GmbH, Freiburg, Germany). Restriction digestion of the insert and vector DNA (pLF plasmid with P_AprE_ promotor, SP_PhoD_ signal peptide, methionine as start codon, and a C-terminal his_10_-tag) was done using *BssH*II and *Xho*I. The insert and vector fragments were purified from a 1% (*w*/*v*) agarose gel using the GeneJET Gel Extraction kit (Thermo Fisher Scientific, Waltham, MA, USA). Both fragments were ligated using T4 ligase and transformed in *E. coli* XL1-blue. Constructs were checked by sequencing (plasmid map, see Supplements Figure S2). Competent *B. subtilis* 007 cells were obtained according to the method of Vojcic et al. [34] with modifications. Instead of 0.025 or 0.01% Cas-amino acids, the respective amount of peptone from casein was used. Pro-PGF was produced in baffled shake flasks. Therefore, two consecutive precultures (5 and 30 mL) were cultivated in sucrose seed medium (sucrose, 40 g L^−1^; soy peptone, 30 g L^−1^; KH_2_PO_4_, 6 g L^−1^; MgCl_2_ ∙ 6 H_2_O, 2.04 g L^−1^; neomycin, 12.5 µg mL^−1^) [35] for 16 and 12 h, respectively, at 37 °C and 120 rpm. For the main culture, sucrose fermentation medium (sucrose, 70 g L^−1^; soy peptone, 50 g L^−1^; KH_2_PO_4_, 5 g L^−1^; MgCl_2_ ∙ 6 H_2_O, 3.06 g L^−1^; neomycin, 12.5 µg mL^−1^) [35] was inoculated with preculture II to an OD_600_ of 0.1 and incubated at 30 °C and 95 rpm. OD_600_ and pH were followed during cultivation. Additionally, samples were taken regularly and centrifuged (20,000× *g*, 10 min, 4 °C). The supernatant (1 mL) was desalted into Tris buffer (100 mM, pH 8.0) using PD midi-Trap G-25 desalting columns (Cytiva Germany GmbH, Dreieich, Germany) to determine PG activity and the protein content according to Bradford [33] and to perform SDS-PAGE as described by Horstmann et al. [16]. The supernatant of the cultivation was harvested by centrifugation (13,700× *g*, 30 min, 4 °C) and phenylmethylsulfonylfluoride (PMSF, 10 mM) was added to inhibit extracellular peptidases. PGF was partially purified, concentrated and desalted by ultrafiltration using VivaFlow 50 filtration cassettes with a polyether sulfone membrane (Sartorius AG, Göttingen, Germany) at 4 °C. A molecular weight cut-off (MWCO) of 100 kDa was used to separate PGF from other extracellular compounds and a MWCO of 10 kDa was used to concentrate and desalt the PGF. Tris buffer (100 mM Tris, 300 mM NaCl, pH 8.0) was used as exchange buffer. The concentrated PGF was stored in 20% (*v*/*v*) glycerol at 4 °C. Azocasein (2.5 g L^−1^) was used as a substrate to investigate residual peptidase activity from *B. subtilis* 007 extracellular peptidases as described by Horstmann et al. [21].

### 2.5. Determination of PG Activity

The PG activity was determined using reversed-phase high-performance liquid chromatography (RP-HPLC) as described by Horstmann et al. [17]. Briefly, the assay was done in a volume of 200 µL at 35 °C in 100 mM Tris, 300 mM NaCl, pH 8.0 (PGF), at 50 °C in 100 mM MES, pH 5.5 (PGB), or at 50 °C in 50 mM sodium potassium phosphate buffer, pH 7.0 (PGC). As a substrate, 20 mM Z-Gln-Gly-OH (Bachem Holding AG, Bubendorf, Switzerland) in dimethylsulfoxide (DMSO) was used for all three PGs. The reaction was stopped by the addition of 20 µL stopping reagent (mercury chloride, 100 mM; hippuric acid, 5 mM). After incubation at 4 °C for 5 min, samples were centrifuged (20,000× *g*, 5 min, 4 °C) to remove precipitated proteins. The analysis (5 µL sample volume) was done on a Knauer Platin-Blue HPLC system equipped with a C18 column (Eurosil Bioselect 300-5, Knauer GmbH, Berlin, Germany) and a diode array detector (DAD). The absorbance at 205 nm was measured. One kat of PG activity was defined as the production of 1 mol Z-Glu-Gly-OH per second.

### 2.6. Determination of Deamidation Kinetics and Protein Solubility

PGC, PGB, and PGF were used in varying activities on gluten, oat, sunflower, broad bean, PPI, and PPC suspensions to determine deamidation kinetics. Afterwards, protein solubility was determined at maximum degree of deamidation (DD(max)). Therefore, 10 mL of plant protein suspensions (62.5 g_protein_ L_tap water_^−1^) of gluten, oat, sunflower, broad bean, PPI, and PPC were prepared under continuous stirring on a magnetic stirrer in tap water. The pH was not adjusted and ranged from pH 6.6 to 7.2 depending on the protein powder used. The suspensions were distributed into Eppendorf tubes, where 320 µL of plant protein suspensions (62.5 g_protein_ L_tap water_^−1^) were incubated for 5 min at 35 °C (PGF) or 50 °C (PGC, PGB) and 800 rpm. Afterwards, 80 µL of the respective PG with an activity of 0.15–4.05 nkat_Z-Gln-Gly-OH_ mL^−1^ was added to start the reaction. This led to a final concentration of 50 g_protein_ L_tap water_^−1^ and 0.03–0.81 nkat_Z-Gln-Gly-OH_ mL^−1^ PG activity. Samples were incubated for 2 h at 50 °C (PGC, PGB) and 3 h at 35 °C (PGF). The deamidation reaction was stopped by heat inactivation in a water bath (95 °C, 5 min), samples were centrifuged (20,000× *g*, 5 min, 4 °C) and the released ammonia was determined using the ammonia assay kit (rapid) (Megazyme Ltd., Wicklow, Ireland).

The degree of deamidation (DD) was defined as the ratio of ammonia released by PG deamidation to the total of released ammonia by chemical deamidation [36]. The total amount of released ammonia was determined by suspending 50 mg of each protein in 1 mL of HCl (2 M) and incubation for 3 h at 95 °C [36,37]. The concentration of free ammonia was determined using the ammonia assay kit (rapid) (Megazyme Ltd., Wicklow, Ireland). The maximum degree of deamidation (DD(max)) was defined as the DD at which no further increase in DD was recognizable. To reach DD(max) in all deamidations, the reaction described above was used with the following modifications. For PGB, 2 h, 50 °C and 0.81 nkat_Z-Gln-Gly-OH_ mL^−1^; for PGC, 2 h, 50 °C and 1.62 nkat_Z-Gln-Gly-OH_ mL^−1^; and for PGF, 4 h, 35 °C and 0.4 nkat_Z-Gln-Gly-OH_ mL^−1^ were chosen.

To determine the increase in protein solubility, the protein content in the supernatant of protein suspensions without the addition of PG and at DD(max) were determined according to Bradford using bovine serum albumin as a reference protein [33]. The increase in protein solubility was defined as the percentage increase in the amount of soluble protein at DD(max) in relation to the protein solubility without deamidation.

### 2.7. Large Scale Deamidation of Oat and Sunflower Protein

To investigate the influence of protein deamidation on the foaming and emulsion properties of oat and sunflower protein, large scale deamidations (500 mL) were done. Therefore, 400 mL sunflower or oat protein powder suspensions (62.5 g_protein_ L_tap water_^−1^) were prepared in a beaker under continuous stirring at 20 °C, transferred into a double-jacket vessel and preincubated for 35 min to reach 35, 40, or 50 °C for deamidation with PGF, PGC, and PGB, respectively. A total of 100 mL of each PG solution, desalted into tap water, was added to reach a total PG activity of 200 nkat_Z-Gln-Gly-OH_ for PGB and PGF or 405 nkat_Z-Gln-Gly-OH_ for PGC. This resulted in protein powder suspensions of 50 g_protein_ L_tap water_^−1^. Incubation was done for 3 h at 50 °C for PGB, 5 h at 40 °C for PGC and 4 h at 35 °C for PGF to reach DD(max) in all approaches. The deamidation approaches were stirred using a magnetic stirrer, and pH and temperature were monitored during the incubation. As a control, untreated protein powder suspensions (50 g_protein_ L_tap water_^−1^) without addition of PG and without incubation (20 °C) were used.

The enzymes in all samples were inactivated by a heating step (5 min, 95 °C, core temperature) in a water bath. The heating phase lasted 35 min in total. The samples were then treated sterilely to prevent falsification of the results by microbial contamination. The samples were centrifuged at 6000× *g* for 10 min at 4 °C and stored at 4 °C. Samples were taken after preincubation and after the heating step to determine protein concentration and DD. Furthermore, as changes in techno-functional properties of proteins could also be caused by protein hydrolysis, the degree of hydrolysis (DH) was determined.

DH was determined using *o*-phthaldialdehyde (OPA) and measuring the absorbance at 340 nm [38]. The samples of oat and sunflower protein at DD(max) were used for the determination of free primary amines. To calculate the DH, the following equation was used.

(1) $$DH=\frac{h}{h_{total}}\cdot 100\%,$$

where h is the number of hydrolyzed peptide bonds and h_total_ is the total number of peptide bonds per protein equivalents. To calculate h_total_, the following equation was used.

(2) $$h_{total}=\frac{c_{protein}}{MW^{*}}$$

where c_protein_ is the total concentration of protein in the sample (50 g_protein_ L_tap water_^−1^) and MW* is the average molecular weight of all proteinogenic amino acids of oat protein (130 g L^−1^, calculated using the amino acid profile by Pettersson et al. [39]) and sunflower protein (112 g mol^−1^, calculated using the amino acid profile by Zhang et al. [40]).

### 2.8. Determination of Foaming and Emulsion Parameters of Oat and Sunflower Protein

The maximum foam volume (V_F_(max)), foam half-time (t_1/2,foam_), foam bubble diameter and emulsion half-time (t_1/2,emulsion_) of the untreated and deamidated oat and sunflower protein suspensions were investigated. Foaming parameters were determined using the set-up described by Ewert et al. [41]. Three double-jacket cylinders (4 cm diameter) with aeration from the bottom were used. Next, 50 mL of sample was applied to the column and tempered to 20 °C. Aeration was done with 300 mL min^−1^ air for 20 s and 1 bar, and pictures of the formed foam were taken immediately after aeration and in 2 min intervals using a Nikon D700 SLR camera with a Nikkor 24–85 mm f/2.8–4.0 AF lens and the software Camera Control Pro 2 (Nikon, Tokyo, Japan). The Tracker Video Analysis Tool V6.2.0. [42] was used to track the decrease in foam volume over time. V_F_(max) was defined as the maximum foam volume present immediately after aeration and t_1/2,foam_ was defined as the time needed to decrease the foam volume to ½ V_F_(max). Pictures were taken until less than ½ V_F_(max) remained. Furthermore, the change in foam bubble diameter was observed by taking a microscopic picture (1000× magnification, Bysameyee, Ningbo, China) directly and 30 min after aeration at the foam–liquid interface.

For the determination of t_1/2,emulsion_, oil in water emulsions were prepared. Using a SilentCrusher M with a 12 F/M nozzle at 20,000 rpm for 6 min (Heidolph Instruments, Schwabach, Germany), 80% (*w*/*w*) continuous phase stained with 20 mg L_H2O_^−1^ methylene blue and 20% (*w*/*w*) disperse phase (miglyol 812 N^®^, Caesar & Loretz GmbH, Hilden, Germany) stained with 5 mL L_miglyol_^−1^ red pepper extract (30 TFE, Gewürzmüller GmbH, Stuttgart, Germany) were dispersed. Emulsion stability was determined in triplicate by applying 50 mL of emulsion to measuring cylinders and taking pictures in 10 min intervals for up to 23 h with the camera set-up described above. t_1/2,emulsion_ was defined as the time needed for the continuous phase to reach ½ of the maximum continuous phase (80% (*w*/*w*)).

### 2.9. Statistical Analysis

Data was evaluated using Microsoft Excel 2022 (Microsoft Corporation, Redmond, WA, USA) and Matlab R2024b(The MathWorks Inc., Natick, MA, USA). All experiments were done in triplicates if not stated otherwise. Standard deviations in protein biochemical experiments were below 5% and below 10% in foaming and emulsion experiments. Statistical analyses were performed using one-way analysis of variance (ANOVA), followed by Tukey’s honestly significant difference multiple-comparison tests. Differences were considered statistically significant at *p* < 0.05.

## 3. Results and Discussion

### 3.1. Production of PGF and PGB

For enzymatic deamidation of different plant proteins and for comparison with commercial PGC, PGB and PGF were produced in *B. subtilis* 007 and *E.coli* BL21(DE3), respectively. Both *B. subtilis* 007 and *E. coli* BL21(DE3) were investigated to produce PGF and PGB, and the expression host with higher yields after downstream processing was chosen. PGF was recombinantly produced by secretion in *B. subtilis* 007 (course of cultivation, see Supplemental Figure S3). Extracellular peptidases cleaved the pro-sequence thereby activating PGF. The cleavage of the pro-sequence was verified by MS (see Supplemental Figure S4). After concentration and partial purification by cross-flow filtration (100 kDa and 10 kDa MWCO), a total PGF activity of 1170 nkat_Z-Gln-Gly-OH_ (63 nkat_Z-Gln-Gly-OH_ mg_protein_^−1^), a yield of 63% and a purification factor of 32 were obtained (see Supplemental Table S3). In comparison, the production of PGF in *E. coli* BL21(DE3) and activation with trypsin yielded 124 nkat_Z-Gln-Gly-OH_ mg_protein_^−1^ [17] and 23 nkat_Z-Gln-Gly-OH_ mg_protein_^−1^ [43] in other studies. Although the specific activity of *B. subtilis* production was lower compared to *E. coli* production, the secretory production in *B. subtilis* allows for easier purification as no chromatography is required.

PGB was produced intracellularly in *E. coli* BL21(DE3) yielding 0.6 µkat_Z-Gln-Gly-OH_ L_culture_^−1^ (see Supplemental Figure S5). After activation by trypsin hydrolysis and partial purification by Ni-affinity chromatography, a total PGB activity of 1166 nkat_Z-Gln-Gly-OH_ (48 nkat_Z-Gln-Gly-OH_ mg_protein_^−1^), a yield of 75%, and a purification factor of 51 was obtained (see Supplemental Table S4). This is in accordance with Horstmann et al., who reported 31 nkat_Z-Gln-Gly-OH_ mg_protein_^−1^ [17]. PGB was also produced recombinantly and secreted in *B. subtilis* 168, 007 and RIK1285, yielding up to 11.8 µkat_Z-Gln-Gly-OH_ L_culture supernatant_^−1^ [21]. Furthermore, PGB was also integrated into the genome of B. subtilis 007, yielding 9.5 µkat_Z-Gln-Gly-OH_ L_culture supernatant_^−1^ [22].

### 3.2. Deamidation Kinetics of Different Plant Proteins

**Figure 1:**
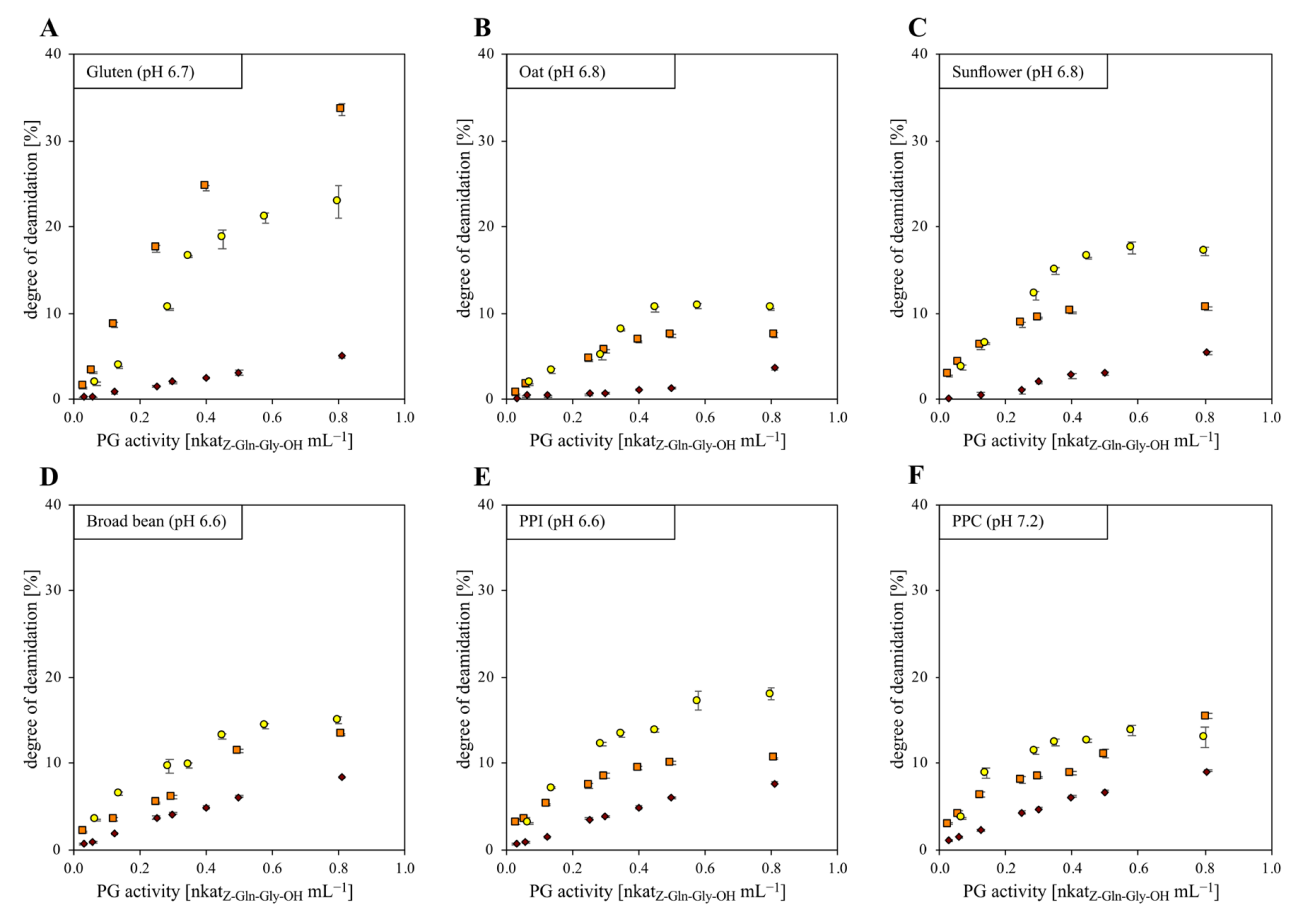
Degree of deamidation achieved by deamidation with PGC (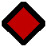), PGB (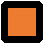) and PGF (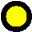) of (**A**) gluten, (**B**) oat, (**C**) sunflower, (**D**) broad bean, (**E**) pea protein isolate (PPI) and (**F**) pea protein concentrate (PPC) (50 g_protein_ L_tap water_^−1^). Conditions: PGC (50 °C, 2 h); PGB (50 °C, 2 h); PGF (35 °C, 3 h).

The partially purified PGF, PGB, and the commercial PGC were used to determine the deamidation kinetics for various plant proteins. Subsequently, suitable conditions for foaming and emulsion experiments were determined based on the deamidation kinetics. The deamidation kinetics were determined under conditions suitable for each PG—PGC, PGB (50 °C, 2 h) and PGF (35 °C, 3 h)—as determined by preliminary experiments. The results for the six plant protein substrates—gluten, oat, sunflower, broad bean, PPI, and PPC (50 g_protein_ L_tap water_^−1^)—are shown in Figure 1.

The deamidation profiles of oat, sunflower, and the legume proteins broad bean, PPI, and PPC showed comparable trends, reaching final DDs between 7.4 and 18.0%. In general, PGF treatment resulted in the highest increase in DD, whereas PGC consistently led to the lowest increase. In contrast, gluten showed the highest DD with PGB (33.5%), compared with 22.9% for PGF and 4.9% for PGC. Overall, the achieved DD depended on the specific combination of PG and substrate. This may reflect differences in the accessibility of L-glutamine residues. The crystal structure of PGC reveals a narrow catalytic pocket containing the catalytic triad (Cys–His–Asp) [13], suggesting that surface-exposed L-glutamine residues are preferentially deamidated. Consequently, the accessibility and position of deamidated L-glutamine residues are critical for the modification of techno-functional properties. The differences in DD between the PGs may additionally be influenced by inhibition due to ammonia formation. Horstmann et al. demonstrated that PGC is more strongly inhibited by ammonia than pro-PGB [16].

Horstmann et al. reported higher DDs of 44% and 46% for 5% (*w*/*v*) gluten suspensions deamidated with 0.5 nkat_Z-Gln–Gly–OH_ mL^−1^ PGC or pro-PGB for 24 h at 40 or 50 °C, respectively [16]. Other studies reported DDs of up to 72% using 2.2 nkat_Z-Gln–Gly–OH_ mL^−1^ PGC for 30 h at 40 °C [28]. Similarly, deamidation of oat protein (1% (*w*/*v*)) in sodium phosphate buffer (pH 7.0) using 1.1 nkat_Z-Gln–Gly–OH_ mL^−1^ PGC resulted in a DD of 50% after 12 h at 40 °C [25]. In the present study, oat protein suspensions deamidated with 0.8 nkat_Z-Gln–Gly–OH_ mL^−1^ reached lower DDs of 7.4%, 3.6%, and 10.5% for PGB, PGC, and PGF, respectively. This discrepancy can be attributed to differences in reaction conditions. In previous studies, plant protein suspensions were prepared in buffered systems adjusted to the optimal pH for PGC or pro-PGB activity. In contrast, suspensions in the present study were prepared in tap water without pH adjustment or added buffer salts, resulting in pH values between 6.6 and 7.2. While this pH range overlaps with the reported optimum of PGC (pH 6.0–8.0) [16], it deviates from the optima of PGB (pH 5.5–6.0) [16] and PGF (pH 8.5–9.5) [17]. Moreover, the absence of buffering capacity may have reduced PG, and especially PGC, stability compared to buffered systems. In summary, the DD is governed by both the type of PG and the substrate characteristics, as well as the protein concentration in the suspension.

### 3.3. Influence of Enzymatic Deamidation on Protein Solubility

**Figure 2:**
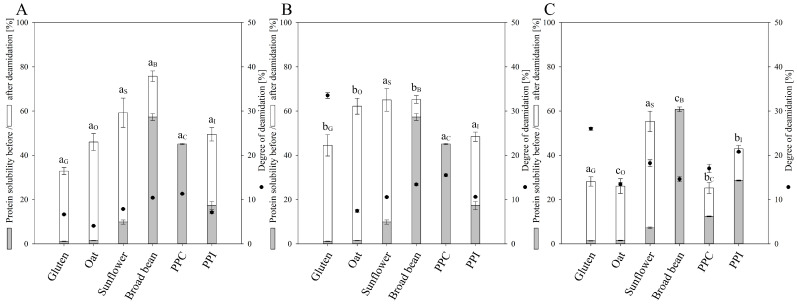
Increase in protein solubility of plant protein suspensions (50 g_protein_ L^−1^ _tap water_) at maximum enzymatic deamidation with (**A**) PGC, (**B**) PGB, and (**C**) PGF. Conditions: PGC (1.62 nkat_Z-Gln-Gly-OH_ mL^−1^) and PGB (0.81 nkat_Z-Gln-Gly-OH_ mL^−1^): 2 h at 50 °C; PGF (0.40 nkat_Z-Gln-Gly-OH_ mL^−1^): 4 h at 35 °C. PPI: pea protein isolate; PPC: pea protein concentrate. Different letters indicate statistically significant differences (*p* < 0.05) in the increase in protein solubility among PG treatments for the same protein (identified by the index).

The increase in protein solubility of the plant protein substrates was determined at the apparent DD(max) (see Figure 2). Therefore, extended incubation times and increased PG activities were examined to verify that the measured DD corresponded to DD(max). Suitable incubation conditions for each PG were selected accordingly. Gluten, oat, and sunflower protein showed low initial protein solubility, which was significantly increased by deamidation with all PGs. In contrast, the legumes broad bean, PPC, and PPI showed higher initial solubility between 17 and 57% at 50 °C and only showed low increases in protein solubility through deamidation (between 0 and 32%). This is in accordance with literature, where legumes, dependent on the powder preparation method, showed high solubility without deamidation [7].

In gluten suspensions, PGB deamidation achieved the highest DD(max) (33.5 ± 0.7%), followed by PGF (26.0 ± 0.3%) and PGC (6.6 ± 0.1%). These deamidations led to an increase in protein solubility of 43 ± 5%, 27 ± 2% and 32 ± 2% by PGB, PGF and PGC, respectively. For oat and sunflower protein suspensions, the highest DD(max) values were obtained through PGF treatment (13.5 ± 0.4% and 18.2 ± 0.8%, respectively). However, the greatest increases in protein solubility were observed after PGB treatment, reaching 61 ± 4% for oat and 55 ± 5% for sunflower protein, compared to 25 ± 2% and 48 ± 3% through PGF treatment. PGC treatment also increased solubility (45 ± 4% for oat and 49 ± 7% for sunflower protein), while again yielding the lowest DD(max) values (4.1 ± 0.1% and 7.9 ± 0.1%). Studies on gluten [28] and PPI [31] have shown that the largest increase in protein solubility is not necessarily associated with the highest DD. In the case of PGB, PGC, and PGF, differences in the tertiary structure of the PGs, including the architecture and width of the catalytic pocket, may influence substrate recognition and the specific glutamine residues targeted for deamidation. Consequently, the location of the deamidated residues may differ between the enzymes, leading to different effects on protein–protein interactions, aggregation behavior, and ultimately protein solubility [28,31].

Overall, these results demonstrate that increased protein solubility is not directly correlated with a high DD. Instead, other factors like substrate specificity or the position of deamidated glutamine residues may be more critical determinants of solubility enhancement.

Previous studies reported higher DD values accompanied by comparable solubility increases, such as a 51% increase in gluten solubility at 70% DD using PGC [29] and a 56% increase in oat protein solubility at 59% DD [25]. In this study, the protein solubility could be increased in all cases except for PPC in case of PGC and PGB and broad bean in case of PGF, while lower DD(max) values were achieved. This indicates differences between buffered and unbuffered systems in terms of deamidation efficiency and solubility response.

The solubility increases observed in other studies have been attributed to deamidation-induced changes in protein secondary structure, including a reduction in β-sheet content and an increase in flexible structures such as β-turns, α-helices, and random coils [25,28]. Enhanced structural flexibility promotes interfacial adsorption and unfolding, facilitating cohesive film formation at oil–water or air–water interfaces [44].

Gluten, oat and sunflower protein showed the largest increase in protein solubility upon deamidation, regardless of the used PG. As gluten is a known allergen, sunflower and oat protein were chosen for the 500 mL scale deamidation of plant proteins and the investigation of techno-functional properties.

### 3.4. Modification of Techno-Functional Properties of Oat and Sunflower Protein Suspensions

To determine the foaming and emulsion properties of untreated and deamidated oat and sunflower protein suspensions, 500 mL scale deamidations were done. Temperature, incubation time, and PG activity were chosen to achieve DD(max) in all deamidations. As a control, the techno-functional properties of untreated oat and sunflower protein suspensions were determined (Table 1). Consequently, the untreated control represented the native techno-functional properties of the plant proteins rather than a temperature-matched reference. It should therefore be considered that heat-induced structural changes may have contributed to the observed changes in functionality in addition to enzymatic deamidation. The effects of heating on protein secondary structure, protein solubility and protein aggregation were investigated in other studies finding no or minor effects at mild heating temperatures (40–60 °C) [31,45]. Here, heat treatment of PPI to 45 °C for 24 h had no effect on protein solubility compared to untreated PPI, while deamidation by PGC increased protein solubility [31]. The influence of heating temperature and time on whey protein showed changes in secondary structure, protein aggregation and interfacial adsorption time starting at 70 °C with only minor changes at 40 and 60 °C [45].

The DD reached in the 500 mL scale deamidation follows the same trend as the small-scale deamidations, with PGC showing the lowest DD with 4.9 ± 0.1% (oat protein) and 9.6 ± 0.1% (sunflower protein). PGF shows the highest DD with 19.6 ± 0.4% and 25.4 ± 0.5% in oat and sunflower protein suspensions, respectively. The protein solubility was increased in all cases. In oat protein, solubility was increased to around 70% by PGB and PGC, while PGF treatment only increased protein solubility to 25 ± 2%. In sunflower protein, PGB and PGF treatment increased protein solubility to around 65%, while PGC treatment only led to 49 ± 1%.

The techno-functional properties of proteins like oat protein can be modified by increasing the protein solubility, which can be achieved not only by deamidation but also by protein hydrolysis [6,46,47]. Enzymatic hydrolysis of sunflower protein isolate to 10% or oat protein to 3% was shown to increase the foam capacity and decrease the foam stability [6,46,47]. Therefore, protein hydrolysis and the DH are of importance for the investigation of the techno-functional properties of plant proteins. To determine the influence of potential protein hydrolysis on the measured parameter, the DH was investigated in the deamidated oat and sunflower protein suspensions. The DH was low in PGC and PGB samples with 1.0 to 1.6%. In comparison, PGF showed the highest DH with 2.2 ± 0.1% and 4.4 ± 0.7% in oat and sunflower protein suspensions, respectively. While the higher DH could indicate proteolytic activity of PGF, the production of PGF involved the activation of pro-PGF by extracellular *B. subtilis* peptidases. The peptidase activity after cross-flow filtration was 72.3 ± 0.5 ΔA·(h·mL)^—1^. Therefore, the higher DH is likely caused by residual peptidase activity after cross-flow filtration. With 4.4 ± 0.7%, the DH in this study was lower compared to the DH reported in the literature, which altered the techno-functional properties of sunflower [6,46,47]. However, the influence of protein hydrolysis on techno-functional properties in this case cannot be ruled out. In contrast, no residual trypsin activity was detected in the PGB preparation using H-Lys-*p*NA and azocasein as substrates following Ni-affinity chromatography.

The emulsion half-lives (t_1/2,emulsion_) of oat protein and sunflower protein are shown in Table 1. While oat protein deamidation by PGC and PGB only increased t_1/2,emulsion_ to 56 and 49 min, respectively, compared to 40 min in the untreated sample, PGF deamidation led to an increase in t_1/2,emulsion_ to 100 min. Hence, protein deamidation using PGF (to a DD(max) of 19.6%) led to a 2.5-times more stable oil-in-water emulsion. In another study, oat protein emulsion properties were also increased by PGC deamidation (59%DD) [25]. However, preparation of emulsion greatly differed between Jiang et al. [25] and this study, as Jiang et al. [25] used 5% (*w*/*w*) rapeseed oil and two homogenization steps while this study used 20% (*w*/*w*) miglyol and one homogenization step. In sunflower protein samples, the initial t_1/2,emulsion_ was higher (220 min) compared to oat protein (40 min). This is in accordance with other studies where sunflower proteins were described to form stable emulsions [48]. PGC and PGF treatment increased t_1/2,emulsion_ to ~340 min, and PGB treatment led to an increase to 686 min. Hence, the deamidation of sunflower protein to a DD(max) of 12.1% using PGB led to a 3-fold increase in t_1/2,emulsion_, making PGB-deamidated sunflower protein a potential emulsifying agent for industrial applications. In a previous study, protein hydrolysis to a DH of 20–30% also led to a 3-fold increase in emulsion stability [6]. However, high DH and therefore higher amounts of hydrophobic peptides, mostly released from water-insoluble proteins, were found to lead to a bitter taste [49,50]. Thereby, protein deamidation by PG could be an alternative method to increase the emulsion stability without the formation of those bitter peptides.

**Figure 3:**
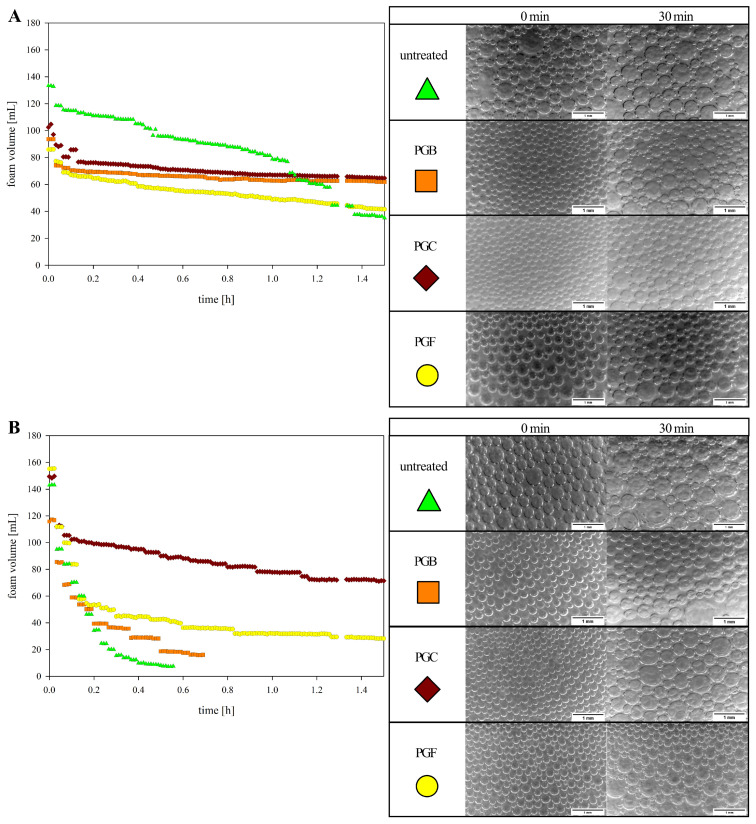
Foaming properties of (**A**) oat and (**B**) sunflower protein suspensions in untreated (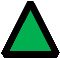) and samples deamidated by PGB (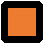), PGC (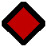), and PGF (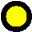). (**A**,**B**) show the decrease in foam volume over time (left) and the foam bubble diameter 0 and 30 min after aeration (right).

Foaming properties of untreated and deamidated oat protein (Figure 3A) and sunflower protein suspensions (Figure 3B) were determined. In case of oat protein deamidation, all PG treatments resulted in comparable maximum foam volumes (V_F_(max)) of ~90 mL, which is a decrease in comparison to the untreated oat protein suspension (134 mL). Meanwhile, foam half-time (t_1/2,foam_) was increased ~ 2-fold in the case of PGC and PGB treatment to 132 and 149 min, respectively, compared to 61 min in the untreated oat protein suspension (Table 1). Meanwhile, no difference in t_1/2,foam_ was observed for PGF. The untreated oat protein suspension showed a continuous decrease in the foam volume over 1.5 h, while PG-deamidated samples showed an initial decline immediately after foaming (Figure 3A, left). However, after the initial decline, the foam stayed consistent over 1.5 h in the case of PGC and PGB (70–80 mL). In contrast, PGF-treated samples showed a continuous decrease in foam volume over 1.5 h, reaching a final volume of around 50 mL, comparable to the untreated sample. Hence, while V_F_(max) is decreased by PGB and PGC treatment, the foam stability as well as t_1/2,foam_ were increased. The change in oat foam bubble diameter at the liquid–foam interface directly after aeration (0 min) and 30 min after aeration is shown in Figure 3A, (right). The initial foam bubble diameter (0 min) of the untreated oat protein suspension was larger in comparison to PGB- and PGC-treated samples, while PGF-treated samples showed a comparable diameter. After 30 min the foam bubble diameter increased in all cases to comparable diameters. These differences in the initial foam bubble diameter correspond to V_F_(max). Furthermore, the smaller bubble diameters also correspond to the higher t_1/2,foam_ of PGB- and PGC-treated samples. The influence of enzymatic deamidation on oat protein foams is not widely studied. Immonen et al. investigated the effects of enzyme aided ultrafiltration of oat protein by PGC in combination with other enzymes on foaming properties [51]. Here, deamidation with PGC led to a decrease in foaming ability, a higher negative zeta potential and maybe higher repulsion between oat proteins at the air–water interface [51]. In case of protein hydrolysis, the untreated oat protein suspension showed faster foaming as well as higher foam stability compared to oat protein hydrolysates [46].

In case of sunflower protein suspensions, the PGB treatment led to a decrease in V_F_(max) to 116 mL compared to the untreated sample with 142 mL. In contrast, after PGF treatment, the V_F_(max) showed an increased value of 167 mL and was comparable to the untreated sample in the case of PGC with 150 mL (Figure 3B). The t_1/2,foam_ increased significantly in the case of PGC treatment (71 min) but only slightly in the case of PGB (9 min) and PGF (8 min) compared to the untreated sunflower protein suspension (4 min) (Table 1). Comparing the decrease in foam volume over time, the results differ from the results of oat protein deamidation.

Untreated sunflower protein foam decreased rapidly and was < 10 mL after 0.5 h. Meanwhile, deamidation with PGC yielded ~80 mL residual foam volume after 1.5 h, thereby yielding higher foam stability compared to untreated sunflower protein. PGB and PGF treatment also increased foam stability, but to a lesser extent than PGC. Sunflower protein showed foam bubble diameters comparable to oat protein at 0 min (Figure 3B, right). After 30 min, however, large foam bubbles formed at the interface of liquid and foam, corresponding to the decreasing foam volume in all samples. The influence of deamidation of sunflower protein on foaming properties is not studied in the literature. Only the hydrolysis of sunflower protein was studied. Here, it was found that high sunflower protein concentrations in combination with low DH led to optimal foaming properties [47].

## 4. Conclusions

This study provides the first comparative evaluation of three PGs, including PGB and PGF, that have not previously been investigated for improving the techno-functional properties of plant proteins. By assessing their performance across a range of industrially relevant plant protein sources under their respective optimal process conditions, this work expands the current knowledge on enzymatic deamidation and demonstrates the application potential of these novel PGs. The solubility of gluten, oat, and sunflower protein increased greatly due to deamidation with the three PGs. In contrast, the legumes broad bean and pea protein showed a lower solubility increase after deamidation; however, they exhibited higher initial protein solubility. In addition, it was found that a greater increase in protein solubility does not correlate with a higher DD(max). These results indicate that protein solubility is not solely determined by the DD. Rather, the specific glutamine residues targeted by the PGs, which may depend on their substrate specificity and catalytic pocket architecture, are likely to have a greater influence on solubility enhancement.

The influence of deamidation on the foaming and emulsifying properties of oat and sunflower proteins was also investigated. The influence of deamidation on foaming and emulsifying properties depended on the substrate–PG combination. Oat protein foam stability was significantly increased by PGC and PGB treatment, while sunflower protein foam stability was significantly increased by PGC treatment. These results demonstrate the complexity of protein deamidation and its influence on different techno-functional properties, which depends on multiple parameters such as the protein substrate, the PG used for deamidation, and the incubation conditions. The aim of this study was to compare different PGs under their respective optimal process conditions while avoiding additional buffer systems to better reflect industrially relevant processing. Consequently, the observed effects represent the combined influence of enzyme-specific reaction conditions and enzymatic deamidation rather than isolated process parameters. Future studies should focus on disentangling these effects under standardized experimental conditions and on elucidating the enzyme-specific deamidation patterns responsible for the observed differences in techno-functional properties. In addition, further validation in industrially relevant food systems or the comparison to commonly used food emulsifiers is warranted.

## Figures and Tables

**Table 1:** Techno-functional properties of oat and sunflower protein (50 g_protein_ L^−1^ _tap water_ suspensions) before (untreated) and after deamidation with PGC, PGB and PGF. DD = degree of deamidation; DH = degree of hydrolysis; t_1/2_ = half-life of foam or emulsion, respectively; V_F_(max) = maximum foam volume. The different letters indicate statistically significant differences (*p* < 0.05) related to the respective protein source.

		Oat				Sunflower			
		Untreated	PGC	PGB	PGF	Untreated	PGC	PGB	PGF
Deamidation conditions	T [°C]	20	40	50	35	20	40	50	35
	t [h]	0	5	3	4	0	5	3	4
	EA [nkat_Z-Gln-Gly-OH_]	0	405	200	200	0	405	200	200
Protein characterization	DD [%]	0.2 ± 0.0 ^a^	4.9 ± 0.1 ^b^	12.4 ± 0.3 ^c^	19.6 ± 0.4 ^d^	0.1 ± 0.0 ^a^	9.6 ± 0.1 ^b^	12.1 ± 1.1 ^c^	25.4 ± 0.5 ^d^
	DH [%]	0 ^a^	1.6 ± 0.1 ^b^	1.3 ± 0.2 ^b^	2.2 ± 0.1 ^c^	0 ^a^	1.3 ± 0.5 ^b^	1.0 ± 0.3 ^b^	4.4 ± 0.7 ^c^
	Protein solubility [%]	2 ± 0.1 ^a^	66 ± 1 ^b^	69 ± 3 ^b^	25 ± 2 ^c^	13 ± 1 ^a^	49 ± 1 ^b^	63 ± 4 ^c^	69 ± 3 ^c^
Techno-functional properties	t_1/2,emulsion_ [min]	40 ± 0.01 ^a^	56 ± 0.01 ^b^	49 ± 1 ^ab^	100 ± 0.01 ^c^	220 ± 14 ^a^	344 ± 7 ^b^	686 ± 35 ^c^	333 ± 9 ^b^
	V_F_ (max) [mL]	134 ± 5 ^a^	88 ± 5 ^b^	94 ± 9 ^b^	85 ± 1 ^b^	142 ± 1 ^a^	150 ± 7 ^a^	116 ± 4 ^b^	166 ± 3 ^a^
	t_1/2,foam_ [min]	61 ± 5 ^a^	132 ± 6 ^b^	149 ± 0.3 ^b^	61 ± 5 ^a^	4 ± 0.3 ^a^	71 ± 9 ^b^	9 ± 0.3 ^a^	8 ± 1 ^a^

## Data Availability

Data will be made available upon request.

## Supplementary Materials

The following supporting information can be downloaded at: https://www.mdpi.com/article/10.3390/foods15152586/s1, Figure S1: Sequence of synthetic gene from native protein-glutamine glutaminase from *Flavobacterium* sp. 316; Figure S2: Constructed plasmid for secretory PGF production; Figure S3: Recombinant production of PGF in *B. subtilis* 007 in shake flasks; Figure S4: MS analysis of PGF purification.; Figure S5: Recombinant production of PGB in *E. coli* BL21(DE3) in a bioreactor; Table S1: Protein, fat and carbohydrate concentrations in protein powders from different plant origins; Table S2: Primers used in this study; Table S3: Purification of PGF from *B. subtilis* 007 cultivation; Table S4: Purification of PGB from *E. coli* BL21(DE3) cultivation.

## Abbreviations

The following abbreviations are used in this manuscript:

PG	Protein-glutamine glutaminase
PGC	*Chryseobacterium proteolyticum* protein-glutamine glutaminase
PGB	*Bacteroides helcogenes* protein-glutamine glutaminase
PGF	*Flavobacterium* sp. protein-glutamine glutaminase
DD	Degree of deamidation
DH	Degree of hydrolysis
V_F_(max)	Maximum foam volume
t_1/2, emulsion_	Emulsion half time
t_1/2, foam_	Foam half time
PPC	Pea protein concentrate
PPI	Pea protein isolate
