# Supplementary material for: Enzymatic Deamidation of Plant Proteins by Protein-Glutamine Glutaminases: Effects on Solubility, Foaming and Emulsion Properties
Source: Foods. 2026 Jul 23;15(15):2586. doi: 10.3390/foods15152586 (PMC13465451; doi:10.3390/foods15152586)
Supplement: Supplementary file 1 [file foods-15-02586-s001.zip › foods-4357697-supplementary.pdf]

Supplements

"Enzymatic Deamidation of Plant Proteins by Protein-Glutamine Glutaminases: Effects on Solubility, Foaming and Emulsion Properties"

Authors

Nicole Roth; Diane Ebinger; Gudrun Horstmann, Lutz Fischer*

University of Hohenheim, Institute of Food Science and Biotechnology, Department of Biotechnology and Enzyme Science, Garbenstr. 25, 70599 Stuttgart, Germany

*Corresponding author: Lutz Fischer

E-mail address: sekretariat-bt@uni-hohenheim.de

Tel.: +49 711 459-23018

Keywords

Techno-functional properties, *Bacteroides helcogenes*, *Chryseobacterium proteolyticum, Flavobacterium*, oat protein, sunflower protein, pea protein, broad bean protein

GCGCGCATGGAACAGGAAAAGAATCCTCAAGAAGTAAATCCACAAACTTCTAATGATTATATAATAGGTCTAATCAAAAA
GTGTGACGATTGTGAAGTTTCAACTTCCAAAACTTTTTTTTATTGTGGAATAAAAGCGGGTGCTTCTTATGTAGAAAATG
ATAGCCCATATCTTAATGATATTAAAGAAAGTATTTCAAACGGAACTCCAATTAAAATTTATTTTGATGAAAATGAGCCA
GAAAGAATAATTTCTGTAGTTAAAATTTCAAATAGTGAAGAAAAGAATTGGAATTTAAATGTGAAATATGATGAAACACC
ATTTAAAACTATTGAGGATTTAAATAGGAAGGTTTCAACTCAAAAAGCTTCTAGTTTTGATTTCACAACAACTGAAGCAG
TCAACTTTTTTAATGCTATGAAAAATAAAAGTTGTGCTATAAACAATCAAAATTTATGTATTCCATTTCAATATGCAAAT
GATGGTTGTTATGCAAGAGCCCACATGATGAGACAACACATGAATTATGCTAGTAAAGATTGTTATAAGATTTTTGCTTA
TGGAAATTTAAAAGTAAATACTTCGTCTACCGGAGTATGTGGTATTGCATGGAGGTATCATGTAGCTCCTTTAATTTCTG
TTAATGGTGTTTGGAATGTAATTGACCCTTCTTTATTTAATCAACCTGTAACCATTACAACTTGGTTGAATAAAATGAAA
TATAACGGAGGTACTGTTGCTACAACAAGTTATCAAAATTCTAGCGTGTATTATTATGATTATGTTTCAAATTATACACA
ATATGATAATAACTATACAGATACTTATAGTACTTTGGCTAATTATAGATATAGACAAACTAGCTGTTCTTTTTAACTCG
AG

Figure S1: Sequence of synthetic gene from native protein-glutamine glutaminase from *Flavobacterium* sp. 316. Orange marks the included restriction sites.

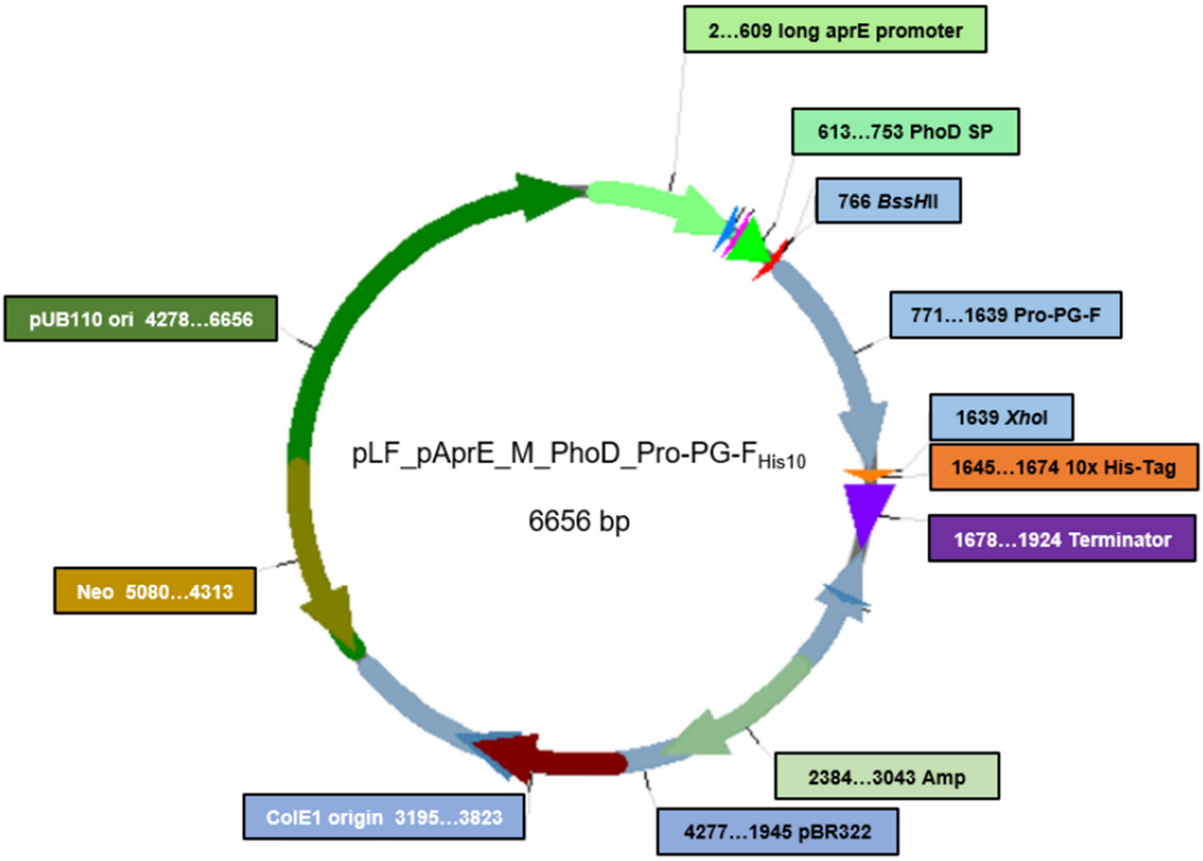

Figure S1: Constructed plasmid for secretory PGF production. Promoter: AprE; start codon: methionine (= M); signal peptide: PhoD; Amp: ampicillin resistance; pBR322: vector backbone; ColE1 origin: origin of replication for *E. coli*; Neo: neomycin resistance; pUB110 ori: origin of replication for *B. subtilis*.

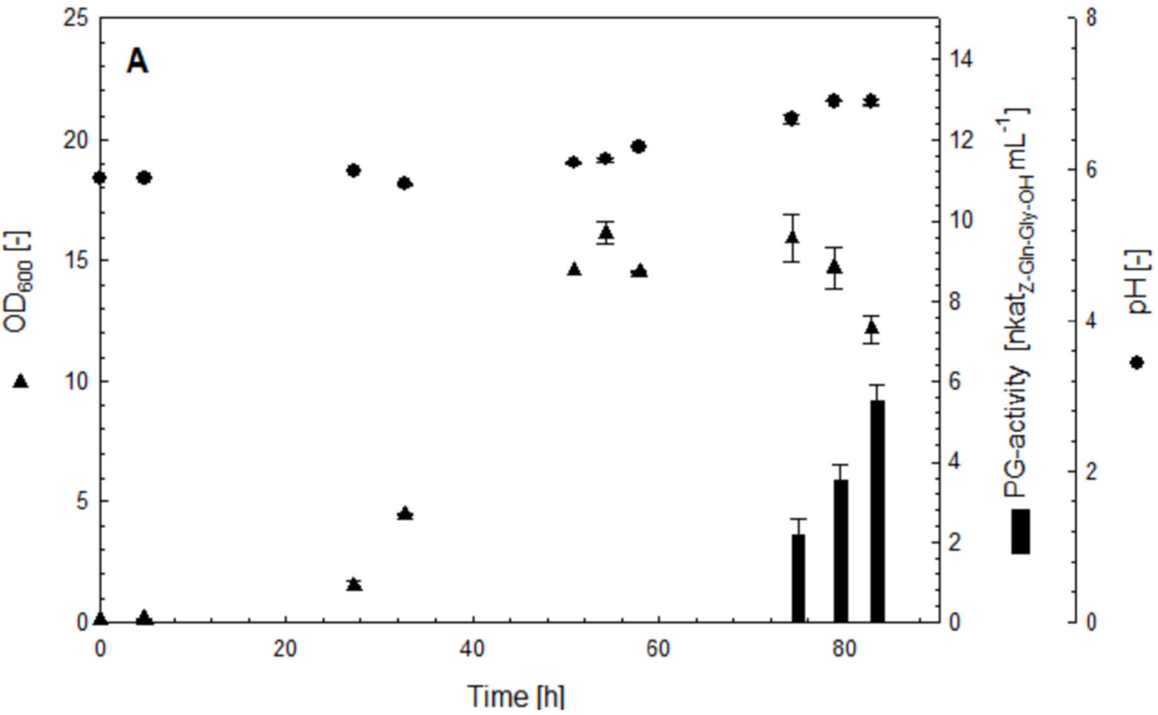

Figure S3: Recombinant production of PGF in *B. subtilis* 007 in shake flasks. Working volume: 0.2 L; 30 °C; sucrose medium.

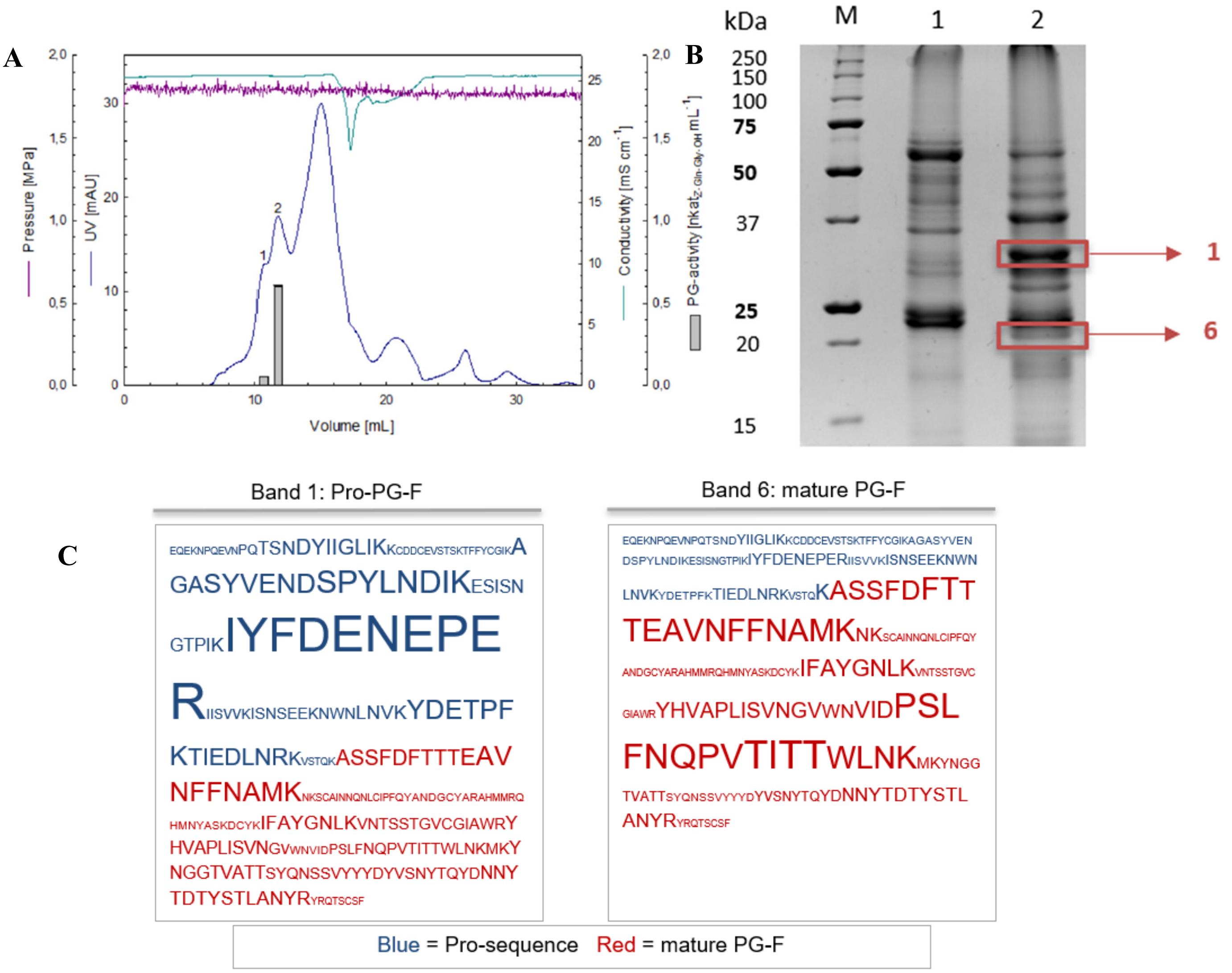

Figure S4: MS analysis of PGF purification. (A) Size exclusion chromatography (SEC) of PG-F. 1 & 2: fractions containing PG-activity; column: Superdex 75 10/300 GL (Cytiva Massachusetts, USA); flow rate: 0.8 mL·min-1; detection at 280 nm. Buffer composition: TRIS (50 mM), NaCl (300 mM), Glycerol (10 %(w/v)), pH 8.0. **(B) SDS-PAGE of SEC elution fractions of PGF. (C) MS results of lanes 1 (Pro-PGF) and 6 (mature PGF) for the identification of the cleavage sites of *Bacillus* peptidases in PGF.**

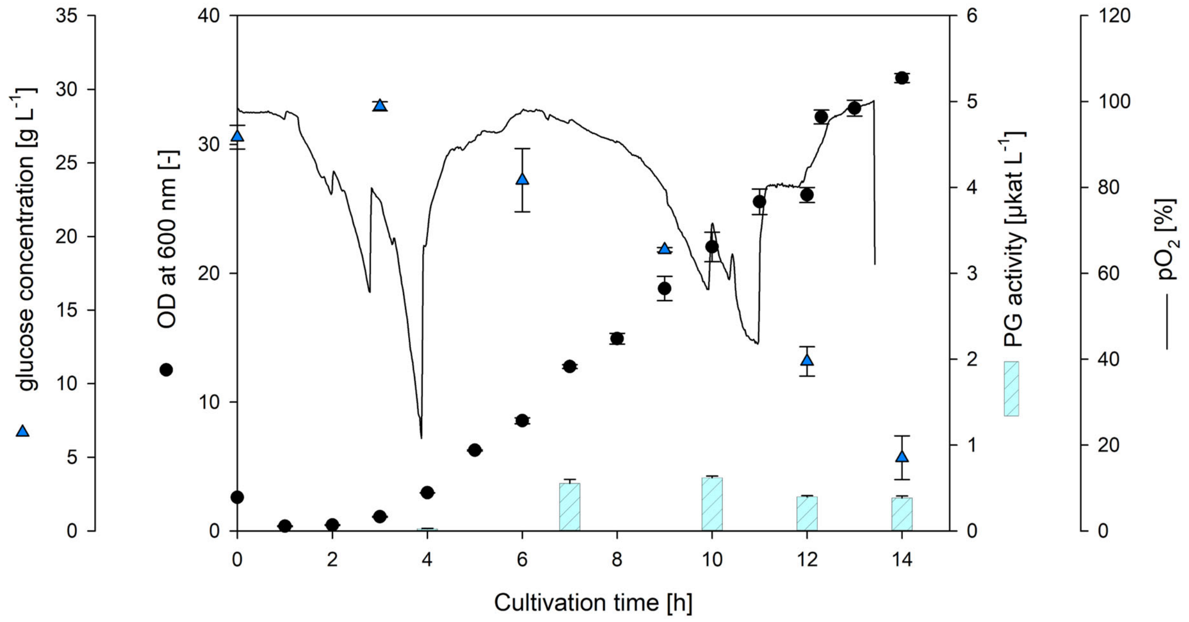

Figure S5: Recombinant production of PGB in *E. coli* BL21 (DE3) in a bioreactor. Working volume: 0.8 L; 30 °C; pH 7.0; 2YT + 3 %(w/v) glucose).

Table S1: Protein, fat and carbohydrate concentrations in protein powders from different plant origins.

Protein source	Protein [%(w/w)]	Fat [%(w/w)]	Carbohydrates [%(w/w)]
Gluten (wheat)	76.0	n.d.	n.d.
Oat	30.8	12.1	23.3
Sunflower	50.0	15.0	12.0
Broad bean	73.0	5.5	n.d.
Pea protein isolate	86.0	4.0	3.2
Pea protein concentrate	55.0	6.0	n.d.

Table S2: Primers used in this study.

Primer	Sequence (5' – 3')	Application
Pro-PG-Fsp_fw_BssHII	CATGCGCGCATGGAACAGGAAAAGAATCC	Amplification of *Pro-PG-F* gene
Pro-PG-Fsp_rev_*Xho*I	GCCTCGAGAAAAGAACAGCTAGTTTGTC	Amplification of *Pro-PG-F* gene
Seq PaprE fw	TCGAGTCTCTACGGAAATAGCG	Sequencing
Seq pLF term rev	CGGATTGAACATGCGGAGGAAG	Sequencing

Table S3: Purification of PGF from *B. subtilis* 007 cultivation. Purification by cross-flow filtration including the separation of target protein from foreign protein by 100 kDa filtration and concentration by 10 kDa filtration.

	**V [mL]**	**PG activity [nkat]**	**Protein [mg]**	**Specific PG activity [nkat/mg$_{Protein}$]**	**Yield [%]**	**PF [-]**
Culture supernatant	330	1855	928	2	100	1
100 kDa permeate	270	1754	70	25	95	13
10 kDa retentate	42	1170	19	63	63	32

Table S4: Purification of PGB from *E. coli* BL21(DE3) cultivation. Purification includes cell disruption by sonification, activation of PGB by trypsin hydrolysis, precipitation of PGB using ammonium sulfate and Ni-affinity chromatography.

	V [mL]	PG activity [nkat]	Protein [mg]	Specific PG activity [nkat/$mg_{Protein}$]	Yield [%]	PF [-]
Crude extract	145	229	1785	0.1	/	/
Hydrolysate	152	1551	1688	0.9	100	1
Salt precipitation	10	1594	156	10.2	103	11
Elution (IMAC)	15	1166	24	47.7	75	51
